# Associations between night-time bedroom environmental factors and objective sleep metrics of older adults

**DOI:** 10.1051/e3sconf/202671605024

**Published:** 2026-06-09

**Authors:** Wan-Tai Au-Yeung, Josephine Lau, Joel Steele, Nora Mattek, Zachary Beattie, Lyndsey Anderson, Allison Lindauer, Miranda Lim, Jeffrey Kaye

**Affiliations:** 1 Oregon Health and Science University, Department of Neurology, Portland, OR 97239, USA; 2 Oregon Health & Science University, Layton Aging & Alzheimer’s Disease Center, Portland, OR 97239, USA; 3 Oregon Health & Science University, Oregon Center for Aging & Technology, Portland, OR 97239, USA; 4 University of Nebraska-Lincoln College of Engineering, Durham School of Architectural Engineering and Construction (DSAEC), Architectural Engineering, Lincoln, NE 68508, USA; 5 University of North Dakota, School of Medicine & Health Sciences, Indigenous Health, Grand Forks, ND 58202, USA; 6 Oregon Health and Science University, School of Nursing, Portland, OR 97239, USA; 7 Vanderbilt University, School of Nursing, Nashville, TN 37240, USA

**Keywords:** Indoor environmental quality, sleep, cognitive status, older adults

## Abstract

Poor sleep quality is associated with adverse outcomes in older adults. Identifying associations between bedroom environmental factors and sleep quality could potentially provide guidance on how to control these factors to improve older adults’ sleep quality. Seventeen older adults with normal cognition (4 males and 13 females, mean [SD] age: 76.6 [8.2] years) and 12 with mild cognitive impairment or dementia (11 males and 1 female, mean [SD] age: 77.1 [8.5] years) living in Portland, Oregon were enrolled in the study. Night-time bedroom environmental factors and sleep metrics were objectively collected over a mean (SD) period of 211 (144) nights in participants’ personal residences. Mixed-effects models were constructed with six sleep metrics (total sleep time [TST], wake after sleep onset [WASO], sleep efficiency [SE], toss-and-turn count, average heart rate, and average respiratory rate) as outcomes and seven bedroom environmental factors (light, noise, temperature, humidity, particulate matter 2.5 [PM_2.5_], total volatile organic compounds [TVOC], and carbon dioxide [CO_2_]) (averaged per night) as predictors including covariates: age, sex, cognitive status, bed-sharing and season. Among all environmental factors, light level had the strongest association with TST (effect size [ES] = −0.051). Relative humidity was most strongly associated with WASO (ES = −0.101) and SE (ES = 0.104). Temperature was most strongly associated with toss-and-turn count (ES = −0.118), average heart rate (ES = 0.052), and average respiratory rate (ES = 0.083), while PM_2.5_ level was strongly associated with TST (ES = −0.050) and SE (ES = −0.058). All reported associations were statistically significant according to the Bonferroni corrected p-value threshold (p < 0.0083). The results provide insight into which environmental factors should be prioritized when attempting to optimize the sleep environment for older adults. Future work will examine whether environmental effects are different on the sleep of older adults with mild cognitive impairment or dementia versus those with normal cognition.

## 1 Introduction

Poor sleep quality is associated with multiple adverse outcomes such as increased risk of chronic diseases, cognitive decline, and mental health problems. Older adults are especially vulnerable to poor sleep quality as sleep quality decreases with age. Sleep quality measured by the Pittsburgh Sleep Quality Index (PSQI) has been shown to be worse in older people[1]. In addition, studies have shown that even healthy older adults may have a measurable decrease in the ability to initiate and maintain sleep, with a reduced proportion of time spent in deeper, more restorative slow-wave sleep and rapid eye movement (REM) sleep[2].

Identifying optimal night-time bedroom environments for sleep in older adults is a low-cost, non-pharmacological way to improve older adults’ sleep quality. The environment can have profound influences on older adults, affecting their quality-of-life domains such as physical, psychological, and social. Limited sleep research studies have focused on older adults in their natural environments as compared to those focused on children or younger adults. Although sleep studies conducted in sleep labs are valuable, the equipment and unfamiliar environment may cause discomfort, and these laboratory studies may not yield reliable information about sleep in the natural environment. Previous studies have examined the influences of environmental factors on older adults’ sleep quality. However, they rarely were examined in combination and measured objectively and longitudinally. In addition, using passive assessment technology to measure sleep metrics may reduce participants’ burden and provide more precise measurements. The objective of this study was to investigate and examine the relative strengths of associations between night-time bedroom environmental quality parameters and sleep quality parameters in older adults.

## 2 Methods

### 2.1 Study Design

This study is an analysis of observational data collected longitudinally in participants’ personal residences.

### 2.2 Study Participants

Participants were recruited from research-ready cohorts from the Oregon Alzheimer’s Disease Research Center (OADRC) Digital Technology Core (DTC) (OHSU IRB #2765) and the Oregon Roybal Center for Care Support Translational Research Advantaged by Integrating Technology (ORCASTRAIT) (OHSU IRB #20236) from 2021–2023. All participants have provided consents to participate in the study either by themselves or by their legal authorized representatives (OHSU IRB #18464 or OHSU IRB #24210).

### 2.3 Data Collection

Sleep outcomes were measured using a commercial bed pressure mat (Emfit, Jyväskylä, Finland) placed underneath the mattress. Our study focused on 6 sleep metrics: i) total sleep time (TST, measured in hours), ii) wake after sleep onset (WASO, measured in minutes), iii) sleep efficiency (SE, % of TST/duration in bed), iv) tossing and turning on the bed (proxied by toss-n-turn counts) and physiological measures, v) average heart rate (Avg. HR, beats/min), and vi) average respiratory rate (Avg. RR, breaths/min). These metrics were chosen to provide a holistic view of the participants’ sleep quality and their physiological wellbeing throughout the nights. Daytime naps and nights with SD recorded as 0 minutes were excluded.

A commercial indoor environmental sensing device (Awair Omni, San Francisco, CA) was placed at shoulder height on the wall of each participant’s bedroom. The device measures light level (lux), noise level (dB), temperature (°C), relative humidity (RH) (%), carbon dioxide (parts per million [ppm]), total volatile organic compounds (TVOC) (parts per billion [ppb]), and particulate matter at 2.5 μm (PM_2.5_) (μg/m^3^) continuously at five-minute intervals.

### 2.4 Data Analysis

Means of the bedroom environmental quality parameters measured from 10pm to 8am were calculated per night except for carbon dioxide. For carbon dioxide, the 95^th^ percentile value was extracted per night. We were interested in learning the steady-state (as approximated by the 95^th^ percentile) carbon dioxide levels, since bedroom occupancy most directly affects carbon dioxide levels in the bedroom. The steady-state method was used to calculate ventilation rates in accordance with ASTM D6245–18 (2023)[3]. Previous research at the University of Nebraska found that the steady-state method was the most reliable for calculating ventilation rates due to its lowest uncertainty[4]. The 95^th^ percentile was used to avoid the outlier while remaining close to the steady-state value[5]. The 95^th^ percentile value would be close to the steady-state concentration when the room was occupied for a long time. Using this value would avoid the underestimation of the carbon dioxide level participants were exposed to.

Since the data comprised repeated nightly measures of sleep outcomes from multiple participants and environmental quality parameters from multiple homes, three-level mixed-effect models were constructed with the 6 sleep metrics as outcomes and all 7 bedroom environmental quality parameters entered simultaneously as predictors while controlling for sex, age, cognitive status, bed-sharing, and season. For sex, female was set as the reference. For cognitive status, normal cognition was used as the reference. Season was set as a categorical variable with Spring being the reference. Random-intercept models were built to find the overall effects of the environmental factors on sleep outcomes.

To compare the relative strengths of associations of the predictors on each sleep outcome, environmental quality parameters were converted to Z-scores, and then the standardized coefficients of the predictors in the mixed-effects models were calculated, which were the effect sizes of the associations between the predictors and the sleep outcomes. The magnitudes of these standardized coefficients were then ranked in each model.

## 3 Results

Table 1 shows the participant characteristics in our sample (n = 29). Twenty-three participants lived in dyadic homes. Twenty-two of these participants shared the same bed, while one slept in a separate room from their partner. One participant who initially shared a bed with their partner transitioned to sleeping alone during the study period. Six participants lived in single-resident homes. Sleep and environmental quality data from 6132 nights were collected overall, with the number of nights from each participant ranging from 20 to 550 and mean (SD) of 211 (144).

Table 2 shows the descriptive statistics of bedroom environmental quality parameters per night over the study period in the homes. It is noteworthy that the noise levels were consistently higher than recommended levels (with mean at 51 dB and min at 42.5 dB)[6]. While the PM_2.5_ levels were generally low, there were nights when the PM_2.5_ levels were at hazardous levels above 300μg/m^3^.

Table 3 shows descriptive statistics for the 6 sleep quality parameters for all 29 participants.

Six mixed-effects models were generated, one for each sleep quality parameter with all environmental factors included simultaneously as predictors while controlling for age, sex, cognitive status, bed-sharing, and season, with random intercept for each participant. Table 4 presents a summary of the relative effect sizes of each of the seven environmental factors on the six individual sleep quality parameters with rankings by magnitude.

Light level was the strongest environmental predictor of TST, with higher light level associated with shorter TST. Light level was also significantly associated with avg. RR.

Higher noise level was significantly associated with longer WASO, and higher toss-n-turn counts as well as lower avg. HR. Noise was the second strongest associative factor with WASO and third with toss-n-turn counts and avg. HR.

For the thermal environment, temperature was the strongest associative factor with toss-n-turn counts, avg. HR, and avg. RR, with warmer environment associated with lower toss-n-turn counts, higher avg. HR, and higher avg. RR. Higher temperature was also associated with higher SE. While RH played the largest role in WASO and SE, with higher RH associated with shorter WASO and higher SE. In addition, higher RH was also significantly associated with lower avg. HR, longer TST and less toss-n-turn counts.

Regarding air quality, PM_2.5_ played a role in participants’ overall sleep quality. As seen from the results, higher PM_2.5_ levels were associated with shorter TST, longer WASO, and lower SE. Higher 95^th^ percentile carbon dioxide level was significantly associated with longer TST, shorter WASO, higher toss-n-turn counts, and higher avg. RR. TVOC only had a statistically significant association with WASO.

## 4 Discussion

### 4.1 Findings in the Context of Previous Research

Light level had a statistically significant inverse relationship with TST, which decreased with higher light levels. This could be due to seasons even though that had been controlled for in the models. Also, participants might not be able to sleep due to other reasons, which resulted in turning on a light in the bedroom which would increase the light level. One caveat regarding our study is that the environmental monitor was mounted on the wall, which may be good at capturing light exposure related to sunlight coming from a window or a bedroom lamp, but poor at capturing light exposure of our participants if they were looking at electronic devices at night.

Higher noise levels were significantly associated with longer WASO, more restlessness (proxied by toss-n-turn counts), and slower avg. HR. The relationships between noise and WASO and restlessness were to be anticipated based on views of how ambient noise may affect sleep hygiene and health. Although there is a relationship between noise levels and cardiovascular health, this relationship may be modified depending on the intensity and chronicity of the noise. No audio allowing for identification of the sound sources was recorded in this study. Thus, it was not possible to pinpoint the type or source of the noise.

The results concerning how temperature affected sleep quality were mixed, with higher temperature significantly associated with higher SE and lower restlessness, but also higher avg. HR and RR. Some past studies have suggested that older adults preferred warmer environments than young adults[7]. This topic merits more research. Temperature effects on HR and RR are well-founded on biological effects. It is of note that the temperature ranges were narrow, constrained by home thermostat settings.

Higher RH was associated with better sleep quality. All the results in this study should be interpreted within the context of the range of environmental quality parameters collected. It is not advisable to have an exceedingly high RH (for example, greater than 65%) as it can cause mold problems in homes. The positive association with RH may be due to participants’ taking showers before bed. Showering could increase the RH in the home with research suggesting that taking a warm shower before bedtime can significantly increase SE. This may be mediated by helping our body to cool down after bathing, which signals to our body that it is time to sleep. This is the so-called “warm bath” effect[8]. RH sensors should be placed in bathrooms in future studies to identify when participants take showers. Also, low RH can potentially lead to dehydration and dry eyes/throats which may disrupt sleep.

The results regarding the effects of PM_2.5_ on sleep quality were consistent with existing literature[9]. Higher PM_2.5_ levels were associated with poorer sleep quality. Higher carbon dioxide levels were associated with more restlessness and higher avg. RR which are also consistent with the existing literature and the biological effects have been well established[10]. However, surprisingly, higher carbon dioxide levels were also associated with longer durations in sleep and shorter WASO, which conflicts with a previous study[11]. TVOC were negatively associated with WASO. TVOC could be from the natural built environment, or it could be from aromatherapy. Some research suggests that some aromatherapies such as lavender aromatherapy could improve sleep[12]. However, we did not collect information regarding whether participants used aromatherapy.

While the focus of this study was on the effects of the physical environment on sleep, we also found that co-sleeping was significantly associated with longer TST, lower avg. HR, lower avg. RR, and higher toss-and-turn counts. There were no significant differences in any sleep metrics between those with normal cognition and those with MCI or dementia. There were significant differences between seasons for TST, avg. HR, avg. RR, and SE. Such differences were consistent with the literature[13].

### 4.2 Limitations

Emfit was found to potentially over-estimate TST in a previous validation study[14]. The mean TST in our study was 8.7 hours (SD 1.8) while a systematic review and meta-analysis of polysomnography studies found that older adults aged 65 or above slept less than 6 hours per night in sleep laboratories[15]. However, the setting for this study is people’s personal residences. Therefore, we cannot make direct comparisons. Sleep diaries may be kept by participants to corroborate the collected objective sleep outcomes. However, in this longitudinal study, we did not require diaries to avoid participant burden (especially for those couples where one individual had cognitive impairment) and lessened ecological validity.

Our study had a small sample size with 29 individuals with 28 of them being white. Therefore, it is not representative of the general population of older adults.

There can be confounding factors that have not been accounted for. For example, though factors contributing to the ambient thermal environment, such as temperature and RH, were objectively measured, we had no concurrent information regarding what blankets may have been used or what clothes they wore when they slept. All these would contribute to the microenvironment in their bed and their subjective thermal sensation. Also, the relationship between the bedroom environmental quality parameters and sleep outcomes can be bidirectional. For example, carbon dioxide levels in the bedroom would be higher when people were awake versus when they were asleep due to breathing rate slowing down significantly during sleep. In this case, it was not the environmental factor influencing the sleep outcomes but the other way around. Another limitation is that the data were only collected in the Portland, OR metropolitan area which only represents one climate.

### 4.3 Future Studies

Future studies should collect data on additional factors that can affect an individual’s sleep quality, such as their body mass index, use of medication, sleep aids, or consumption of caffeine. In this study we measured the overall effects of the environmental factors on sleep quality outcomes while allowing random intercepts. However, everyone may respond to the environment differently based on their characteristics. It will be worthwhile to include random slopes and investigate the interactions between participants’ characteristics and their response to the environment in future studies. This will enhance personalized optimization of bedroom environments to improve sleep quality. In addition, future studies conducted in indoor environments influenced by multiple climates will provide richer data and further improve understanding on the relationship between bedroom environment and older adults’ sleep quality.

### 4.4 Conclusion

There is evidence that indoor environmental quality parameters in the bedroom play a role in older adults’ sleep quality. In this study, we examined the effects of multiple bedroom environmental quality parameters on older adults’ objectively measured sleep outcomes and estimated and ranked their effects. Identifying environmental interventions that can optimize older adults’ sleep is important as older adults are vulnerable to adverse health outcomes related to poor sleep quality. The results of this study will provide useful reference points for bedroom environmental design in the future.

## Figures and Tables

**Table 1.** Participant characteristics

	Mean (SD) or Count (%) (n=29)
Age (years)	76.7 (8.13)
Male	15 (51.7%)
Cognitive status	Dementia: 8 (27.6%) MCI: 4 (13.8%) Normal cognition: 17 (58%)
Race	White: 28 (96.6%) Black: 1 (3.4%)
Years of Education	16.7 (2.82)

**Table 2.** Descriptive statistics of bedroom environmental quality parameters per night over the study period in 18 homes.

	Mean ± SD	Median (IQR)
Light (lux)	4.75 ± 11.43	0.67 (0.02 – 4.23)
Noise (dB)	51.7 ± 2.94	51.4 (50.1 – 52.6)
Temperature (°C)	21.1 ± 2.12	20.6 (19.4 – 22.5)
RH (%)	47.4 ± 7.92	47.5 (42.1 – 52.7)
PM_2.5_ (μg/m^3^)	5.65 ± 21.7	1.64 (0.97 – 2.88)
95^th^ %tile Carbon dioxide (ppm)	839 ± 257	783 (635 – 998)
TVOC (ppb)	294 ± 550	176 (103 – 312)

**Table 3.** Descriptive statistics of sleep metrics per night over the study period for the 29 participants.

	Mean ± SD	Median (IQR)
TST (hours)	8.72 ± 1.79	8.85 (7.67 – 9.98)
WASO (minutes)	27.5 ± 29.2	20.5 (8.50 – 37.5)
SE (%)	90.7 ± 4.93	91.5 (88.6 – 93.9)
Toss-n-turn counts	64.6 ± 40.6	60 (35 – 85)
Avg. HR (beats/minute)	63.5 ± 8.21	63.7 (57.2 – 68.4)
Avg. RR (breaths/minute)	14.5 ± 2.46	14.1 (12.9 – 15.9)

**Table 4.** Standardized coefficients (effect sizes) of the 7 indoor environmental quality parameters on 6 individual sleep outcomes. Relative rankings by magnitude are in parentheses.

	TST	WASO	SE	Toss-n-turn count	Avg. HR	Avg. RR
Light level	−0.051** (1)	−0.011 (6)	0.004 (7)	−0.003 (7)	0.005 (4)	0.015* (4)
Noise level	0.011 (5)	0.073** (2)	−0.019 (4)	0.040** (3)	−0.026** (3)	0.008 (5)
Temp.	−0.001 (7)	0.009 (7)	0.050** (3)	−0.118** (1)	0.052** (1)	0.083** (1)
RH	0.046** (3)	−0.101** (1)	0.104** (1)	−0.029* (4)	−0.043** (2)	−0.006 (6)
PM_2.5_	−0.050** (2)	0.040* (3)	−0.058** (2)	0.024 (5)	0.002 (5)	0.019 (3)
95^th^ %tile CO_2_	0.030* (4)	−0.036* (4)	0.013 (6)	0.086** (2)	−0.002 (6)	0.026** (2)
TVOC	−0.015 (6)	−0.026* (5)	0.016 (5)	−0.004 (6)	0.001 (7)	0.001 (7)

* indicates p-value < 0.05

** indicates p-value < 0.05/6 = 0.0083
